# The Story of Goldilocks and Three Twitter’s APIs: A Pilot Study on Twitter Data Sources and Disclosure

**DOI:** 10.3390/ijerph17030864

**Published:** 2020-01-30

**Authors:** Yoonsang Kim, Rachel Nordgren, Sherry Emery

**Affiliations:** 1 Social Data Collaboratory, Public Health, NORC at the University of Chicago, Chicago, IL 60603, USA; kim-yoonsang@norc.org; 2 Biostatistics, School of Public Health, University of Illinois at Chicago, Chicago, IL 60612, USA; rknordgren@gmail.com

**Keywords:** Twitter, social media data source, point of access, data quality, e-cigarette

## Abstract

Public health and social science increasingly use Twitter for behavioral and marketing surveillance. However, few studies provide sufficient detail about Twitter data collection to allow either direct comparisons between studies or to support replication. The three primary application programming interfaces (API) of Twitter data sources are Streaming, Search, and Firehose. To date, no clear guidance exists about the advantages and limitations of each API, or about the comparability of the amount, content, and user accounts of retrieved tweets from each API. Such information is crucial to the validity, interpretation, and replicability of research findings. This study examines whether tweets collected using the same search filters over the same time period, but calling different APIs, would retrieve comparable datasets. We collected tweets about anti-smoking, e-cigarettes, and tobacco using the aforementioned APIs. The retrieved tweets largely overlapped between three APIs, but each also retrieved unique tweets, and the extent of overlap varied over time and by topic, resulting in different trends and potentially supporting diverging inferences. Researchers need to understand how different data sources can influence both the amount, content, and user accounts of data they retrieve from social media, in order to assess the implications of their choice of data source.

## 1. Introduction

Health and social research using social media data is increasing rapidly [1,2]. Twitter is the most widely used source because of its public-facing nature and relatively straightforward access to data through public APIs. Twitter data have been used for infodemioloy/infoveillance studies, tracking health attitudes and behaviors, and measuring community-level environment related to health outcomes [3,4,5,6,7]. There are multiple ways to access Twitter data. Researchers likely choose one source over another because of its accessibility or affordability. However, there is no systematic guideline to help researchers evaluate the advantages and limitations of each data source for their research question. This problem is not limited to Twitter data; other social media platforms and data vendors also provide insufficient technical guidance to make informed or transparent decisions. The interpretation, validity, and replicability of this study’s findings are directly related to data sources and their credibility.

### 1.1. Our Story and Motivation

Our interest in the sources of social media data stemmed from our first experience with the Twitter streaming API data. Since when we began our social media research in 2012, our main Twitter data source has been the PowerTrack, historic archive of Firehose, which provides access to 100% of the public posts that match the search filter criteria, and offers the advantage of supporting retrospective inquiry. For broad behavioral and public opinion research, idiosyncrasies of slang and regional dialects, as well as unanticipated marketing or policy events, make it challenging to anticipate all potentially relevant search terms ahead of time. Thus, we weighed the cost of the Firehose with the security of complete coverage and the opportunity to go back and retrieve relevant posts missed by our initial search filters. We decided that the Firehose offered us the best opportunity to capture relevant data for our research agenda. Yet, once we had developed a robust set of keyword search filters, we wondered whether the ‘free’ API could provide a comparable sample of data, which would be sufficiently generalizable for our research questions, without the ongoing subscription cost. There was virtually no technical documentation of how the public stream was generated. Thus, we decided to undertake an experiment: a direct comparison of the amount, content, and data quality [8] for each data source.

In this early and rough experiment, we retrieved e-cigarette-related tweets in a two-stage process. We first collected a broad archive of tobacco-related tweets—including various tobacco, e-cigarette/vaping products, related attitudes, behaviors, and policy—using hundreds keyword-based search rules via the PowerTrack. Then, from this broad tobacco archive, we filtered for tweets that matched our e-cigarette search filter (*N* = 82,205). We then took a random sample of 6000 tweets from the tobacco archive, which did not match e-cigarette search filter, and manually labeled those to count number of e-cigarette relevant tweets missed by our search filter; 20 relevant tweets were found among the unmatched sample. For the same time period, our research partner had archived Twitter data pushed from the Streaming API, so-called “spritzer”, which Twitter states provides approximately 1% sample of all tweets in near-real time [9,10]. From the archive of the spritzer data, we used the same search filter to extract 387 e-cigarette-related tweets over the same time period. We noted that this 387 appeared to be far less than what one would expect from a 1% sample—roughly 1% of 82,205 Firehose tweets, but we were also curious of the quality of the data. Again, we took a random sample of 6000 spritzer tweets that were not retrieved by our e-cigarette search filter and found that none was relevant to e-cigarettes.

This seemingly different result using the same search filter raised several questions: was the difference in amount, even after adjusting for the 1% sampling fraction, because the unmatched sample from the Firehose tobacco database was more tobacco-related than a sample of all spritzer tweets? If so, does the spritzer API guarantee an unbiased random sample of full data? Could those 387 e-cigarette tweets be treated as representative of entire e-cigarette-related conversations on Twitter? Further, can we get the same conclusion about e-cigarettes from the two data sets? These questions inspired us to further explore different ways to access Twitter data. Therefore, we designed a “Goldilocks” experiment to compare data outputs of the three main Twitter access points (the Search API, the Streaming API and the Historical PowerTrack) across three levels of amount: a narrow topic, a moderately broad topic, and one more general.

### 1.2. Twitter Data APIs

Using Twitter’s documentation, along with evidence from other researchers, we created a brief summary of each API.

#### 1.2.1. The Streaming API

The Streaming API is the most widely used publicly available source of Twitter data. It is push-based API, meaning data are pushed in from the endpoint data server as tweets are posted. It requires a persistent connection to the data server and constant monitoring [11]. Thus, a robust local infrastructure is necessary to maintain the connection. This API was designed to deliver limited volumes of data by two types of data streams: sampled stream and filtered stream. The sampled stream, called “spritzer”, gives a 1% random sample of all tweets posted [12,13]. The filtered stream pushes tweets that match queries with 1% limit on the amount; when the tweets matching queries exceed 1% of all traffic on Twitter, the 1% cap gets applied, and the API gives a message that indicates how many tweets have not been pushed from the data server. It is, however, unclear how the tweets are selected when the 1% limit is reached. The filtered stream pushes any tweet that contains matching keywords in its text or URLs (using the “track” parameter) [14]. A user can build search filters using a space (meaning AND) or comma (meaning OR), but it does not offer an operator, like a Boolean NOT logic, to exclude certain contents. In addition, there is a rate limit: maximum of 400 keywords and a limited number of user names were allowed at the time of our data collection [15]. Twitter may change the rate limit over time.

There are important disadvantages to using the Streaming API: since it pushes data in near-real time, it is impossible to get tweets posted in the past and thus impossible to capture content related to unanticipated events. Further, if the API connection is down for some reason, there is no way to retrieve the data occurred during the downtime.

#### 1.2.2. The Search API

The Search API is another publicly available, free source of Twitter data. It is a pull-based API, meaning data are pulled by end users. An advantage of the Search API is that it pulls tweets posted in the past ~7 days, but the drawback is it does not guarantee to pull the full amount as Twitter describes it “is focused on relevance and not completeness,” [16] and there exists no clear description what the limit is and how it is applied [12,17]. This API searches tweets that match keywords in the same way Twitter Search does (i.e., as one would search on Twitter.com using keywords). Some operators allowed to build search filters are double quote to match an exact phrase, minus “‒” for exclusion, OR, and hashtag.

An important disadvantage of the Search API is its rate limit. When we were collecting tweets for our experiment, the limit was 180 requests per 15 min window for per-user authentication, and a maximum 100 tweets per request, suggesting a total limit of 18,000 tweets per 15 mins [17,18].

#### 1.2.3. Historical PowerTrack

Historical PowerTrack, operated by Gnip, provides access to all public tweets that matches queries from the archive of Firehose stream. It is job-based API, thus there is no need to maintain a constant API connection. It supports fine-grained queries, called “PowerTrack rules” to set up search filters with user-friendly interface and rule-managing methods [19,20]. The PowerTrack rules enable a complex search filter to search for specific tweets posted in a specified time frame. Historical PowerTrack also provides enhanced metadata such as expanded URLs and more information on user locations. Up to 1000 rules per job and 60 jobs per day are allowed. The primary disadvantage is cost: it can be prohibitively expensive for individual researchers or graduate students to access independently.

### 1.3. What Has Been Done?

The Streaming and Search APIs provide subsets of all tweets posted. It would be ideal for research purposes if the data obtained by these two APIs were sampled randomly from and representative of the full stream. Indeed, Twitter’s forum states that the Streaming API’s sample stream is “a random sample of 1% of the tweets being issued publicly”. Many researchers simply accept the statement and assume that the Streaming provides a random sample of tweets [12,21]. However, there is no documentation by Twitter that describes the sampling frame, or whether their sampling draws the first few tweets in a few min interval or is stratified by any parameter like location or time [22]. Kergl et al. (2014) examined the generation of tweet IDs and discovered that the spritzer stream data consist of tweets generated during a specified 10-milisecond interval for every second, indicating the systematic sampling [23]. However, to the best of our knowledge, it remains unknown whether the same sampling frame is also employed to the filtered stream when the 1% limit takes effect.

A few studies have explored different points of access of Twitter data as to their randomness and limits on the amount. boyd and Crawford (2012) noticed that some public tweets are missing from the Firehose and raised a question about randomness and representativeness of gardenhose data and spritzer data [22]. In a blogpost, Ahmed (2015) compared the Search API and Firehose for tweets mentioning Ebola [24]. He retrieved tweets using three different tools: DiscoverText to retrieve Firehose data, Chorus and Mozdeh to retrieve the Search data. The DiscoverText retrieved the most (*N* = 195,700) Ebola tweets. However, unexpectedly, the amounts of Ebola-related tweets were different between Chorus (*N* = 155,000) and Mozdeh (*N* = 145,300) although the two used the same API. The content looked similar across the three tools based on top frequent words shown in world clouds, although this finding may be explained by the fact that the tweets were pulled using one specific keyword “ebola”.

Morstatter et al. (2013) investigated whether tweets obtained by the Streaming API is a good representation of daily activity on Twitter about conversation around Syria by comparing with the Firehose data [25]. The Firehose data had consistently larger volume during the study period, and the daily coverage of the Firehose data by the Streaming API data varied widely, ranging from <20% to 90%. To study randomness they conducted repeated random sampling of the Firehose data to obtain a plausible distribution of the Jensen–Shannon divergence statistic to measure topic differences. Then they computed the same statistic based on the Streaming data to compare with the distribution obtained from the repeated random samples. They observed that the Streaming API data tended to deviate from the pattern of random samples of the Firehose data, and the Streaming data did worse in finding top hashtags and topics than the majority of the Firehose random samples, especially when the Streaming’s coverage of Firehose data was low.

Gerlitz and Rieder (2013) compared the volume of tweets posted by three bot accounts collected by the Streaming API with their total activity on Twitter and concluded that the amount of data retrieved was similar to what would be expected with random sampling [11]. However, their description lacks important detail, including how they accessed the data on the total activity of the three bots on Twitter, to gauge validity of their conclusion.

Driscoll and Walker (2014) explored the Streaming API’s limit, comparing it with the Firehose (via PowerTrack) as a reference [26]. The authors collected high volume tweets (up to ~250,000 tweets per 15 min window) around 2012 presidential debate using the hashtags *#debate* and *#debates*. The Streaming API retrieved 80% of the PowerTrack data. The Streaming API does not allow filter data by hashtag-matching, thus they collected tweets that match “debate(s)” and then selected tweets that contain “#debate(s)”. This less sophisticated filtering of the Streaming API made it easier to reach the 1% limit for high traffic tweets.

Tromble, Storz, and Stockmann (2017) in their working paper [27] compared data obtained from the Streaming, Search, and Historic PowerTrack APIs on four events—each one based on a single keyword (#jointsession, #ahca, #fomc, @realdonaldtrump). They accessed the PowerTrack data by a third-party tool DiscoverText (http://discovertext.com). Their unique contribution was that they compared characteristics of tweets and users captured by the Streaming and Search APIs with those captured by PowerTrack. In the analyses for a subset of the topics, the tweets by verified users were more likely to appear on the Search data, while less likely to appear on the Streaming data, compared to the PowerTrack data. However, some results were not consistent across the topics explored. The bottom line of their findings was that the Search and Streaming data were not representative of PowerTrack data.

These few studies used Firehose data as a benchmark and found that the public APIs provided access to a subset of full stream data as described in the API documents. However, they also exhibited contradictory findings about randomness and representativeness of the Streaming API data. As a result, questions still remain. Only one study so far has investigated all three sources together [27]. In fact, most studies investigated Streaming by comparing it to Firehose, and data collected by the Search have not been well examined. In addition, the topics of the above studies have narrow scope defined by only one keyword and not broad enough to capture variety of content or user accounts. Further, the above studies collected data for rather a short timeframe—not long enough to assess whether each API provides suitable data for surveillance purposes.

### 1.4. Objectives

We aim to provide guidance for social media researchers about the parameters of different sources of Twitter data, in order to inform both the choice of data source for particular research topics, as well as to support the development of a framework for standard disclosure. This endeavor is necessary because the online documents provided by Twitter lack sufficient detail to enable social media researchers to understand the differences, pros and cons of data APIs, so that one can decide suitable source for specific research questions and know the limitation to make valid inference and conclusion. What matters is that researchers should understand the quality and limitations of the data in hand in order to make valid and robust inferences.

In this study we compare three sources of Twitter data: Gnip Historic PowerTrack API, the Streaming API, and the Search API, to collect data from each about three topics with varying levels of popularity: tweets about anti-smoking, e-cigarettes, and tobacco. These topics were intended to capture small (anti-smoking), moderate (e-cigarettes) and large (tobacco) volume of conversations. Between the three APIs we: (a) compare the amount of tweets, overlapped and unique tweets from each API, (b) examine content, and (c) user accounts of tweets. We hypothesize that the Historic Powertrack—the Firehose—would yield the most tweets across all topics. Ideally the three APIs should give similar results and ultimately consistent conclusions, but based on others’ work and our own experience, we expect that the Streaming and Search APIs may not yield random or representative samples across all topics or consistently over time. We discuss the consequences of using one API over another and strategies for selecting the most appropriate and practical source of social media data. Although we use Twitter data as a use-case in this study, understanding data source and quality is crucial first step for analyzing data from other social media platforms too.

## 2. Materials and Methods

### 2.1. Data

We obtained tweets posted from 15 January to 30 June 2015 via three APIs: Gnip Historic PowerTrack, the Streaming (filtered stream), and the Search. To collect tweets, we used the following keywords, which were curated to mix keywords of high and low volumes.

- Anti-smoking: @drfriedencdc, smokefree, secondhand smoke, quitline(s), #quitnow, cdctips.
- E-cigarettes: ecig, vaper(s), vaping, eliquid(s), e-liquid(s), cartomizer.
- Tobacco: cig, hookah(s), tobacco, shisha, rello(s), cigarillo(s), skoal, snus, marlboros.

These keywords are not comprehensive because we did not aim to capture all the relevant tweets to represent each topic, but rather we aimed to collect comparable data across the three APIs. Also we did not specify or restrict data by languages or geographical regions.

The Streaming: Python module *twitter* was used [28]. Tweets were added into data files as they were pushed to our server. We have not encountered any rate limits although the connection to data server was disrupted several times. The Streaming API retrieved a lot of seemingly irrelevant data, of which tweet content and URLs did not match our search filter.

The Search: Again, the Python module *twitter* was used. The script was executed every 30 mins to pull data that match our search filter. After testing the script, we decided that the 30-min window was small enough to avoid missing out tweets and not too wide to retrieve excessively overlapping tweets between adjacent pulls. We had “over capacity” errors multiple times [29]. This API pulls recent tweets, thus tweets were partially overlapped between adjacent pulls, which is advantageous when short term problems arise. We de-duplicated overlapped tweets based on tweet IDs. To retrieve tweets mentioning the “e-liquid”, the query “e AND liquid” was used instead of “e-liquid” because the Search API uses the operator “–” to indicate exclusion. We selected tweets containing “e-liquid” after the data collection.

Historic PowerTrack: We submitted a request for one month’s worth of data at a time. For example, we requested Gnip in the 3rd week of March to pull tweets posted from 1st to 28th February. Gnip provided the cost and volume estimates. We then accepted the estimates and pulled data via URLs that pointed to data locations in the Gnip server. The data pull request was made six times between February and July of 2015. We will call this API PowerTrack henceforth.

### 2.2. Analysis

To make the data sets from the three APIs comparable, only tweets that contain the keywords in tweet text or URLs were included for analysis. This removed about 1.2 million irrelevant tweets from the Streaming and Search APIs. We computed overall monthly amounts of overlapping and unique data and daily amounts of tweets for each of the three topics across the APIs. For periods when one API retrieved significantly more data than the other two, we examined the content of tweets posted during those days by type of user accounts.

In addition to comparing amounts of tweets across the APIs, we analyzed content and user accounts. Since tobacco is the broadest topic, encompassing various tobacco products, we compared content of tweets across the APIs by comparing the rank of top hashtags in tweets posted on World No Tobacco Day across the APIs using Kendall’s tau. For the less broad topic, e-cigarettes, we examined and compared types of user accounts of tweets across the APIs. A random sample of 1,000 user accounts was pulled from each API and manually labeled for e-cigarette relevance and primary language (English vs. not English) based on their profiles and tweets. Then the user accounts that posted e-cigarette-relevant tweets in English were further labeled for marketing vs. non-marketing based on their profiles and tweets. We defined marketing accounts as those explicitly marketing or promoting e-cigarette/vaping products and paraphernalia, posting information about new products, product reviews, coupons, or deals. For instance, a user that posted “Check out my e-liquid review of [product name]” is considered a marketing account. For the anti-smoking-related tweets, we expect the least diverse content and user types, thus we compared only amounts of tweets.

## 3. Results

### 3.1. Overall

**Figure 1:**
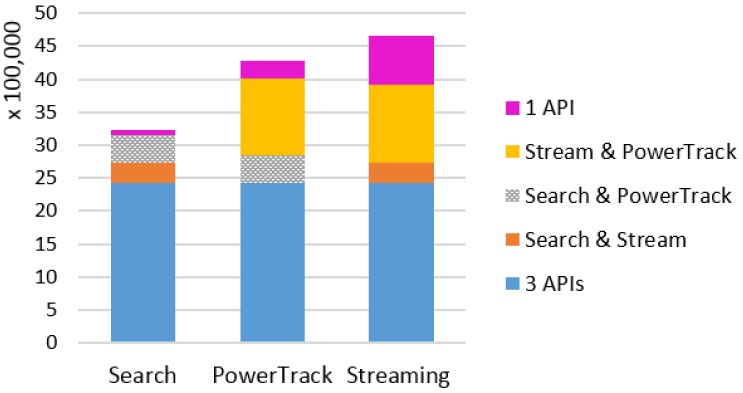
Number of tweets retrieved by each API, combining all three topics. Blue indicates the amount of tweets retrieved by all three APIs, yellow, textured gray, and orange indicate the amount retrieved by two APIs, and magenta indicates the amount of tweets unique to each API.

Combining all three topics, about 5.4 million tweets were collected in mid-January to June via all three APIs combined. The monthly amounts of unique and overlapping tweets from each API are presented in Table A1 in Appendix A. Figure 1 displays the number of tweets retrieved by each API during the study period. The Search API retrieved 3,228,665, the PowerTrack did 4,271,429, and the Streaming retrieved the largest amount of 4,662,372 tweets. The majority of each API data overlaps with one another, either by all three (indicated by blue “3 APIs”) or two APIs (yellow, gray, orange), but each API retrieved unique tweets too (magenta “1 API”). The Streaming API retrieved the largest number of unique tweets—about 750,000—throughout the study period. Especially one quarter of the Streaming data in January was unique to that API alone (Appendix A Table A1). These results raise a question whether PowerTrack, which is an archive of the Twitter Firehose data, may not, in fact, return 100% of relevant tweets, as most researchers believe. Further, we find that the coverage of each API varies from month to month and across topics.

### 3.2. Tobacco

**Figure 2:**
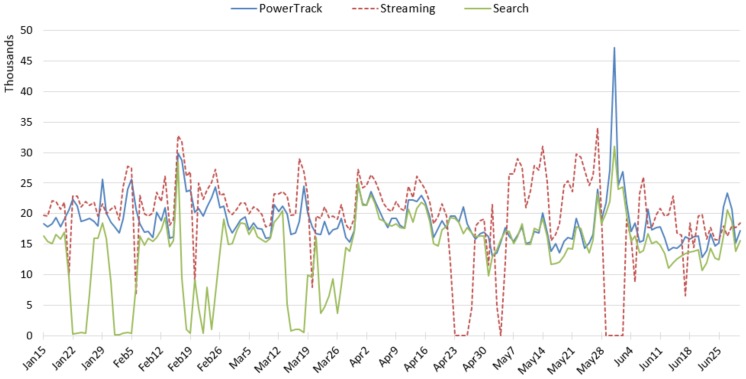
Daily amount of tobacco-related tweets across the APIs.

Amount: The daily counts of tobacco-related tweets by APIs are displayed in Figure 2. As we expected, the tobacco topic retrieved the largest amount of data among the three topics. It is clear that data collection was interrupted multiple times for the Streaming and the Search APIs; we believe this is not uncommon. The Streaming returned zero tweets during the interruptions, but the Search returned a small number of tweets (as small as ~200 a day) despite interruptions, probably because it pulls recent tweets. The Streaming API retrieved the largest number of tweets on most days, followed by PowerTrack, and the Search retrieved the fewest, even when the connection to data server was not interrupted.

**Figure 3:**
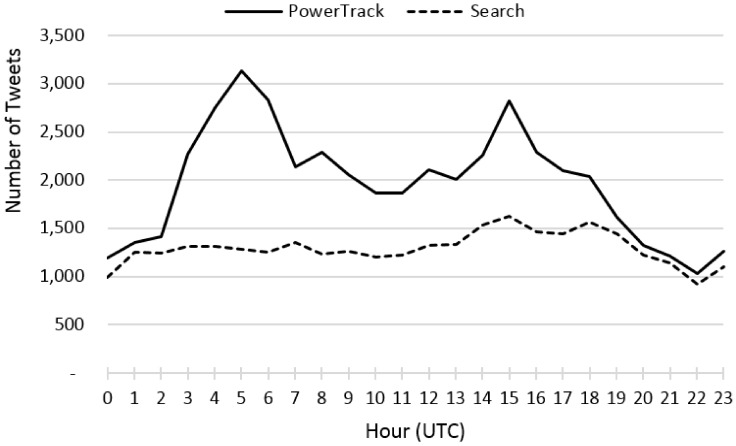
Amount of tweets per hour on World No Tobacco day.

World No Tobacco Day on 31st May stimulated activity of tobacco-related tweets. This outstanding amount was well captured by PowerTrack, and partially by the Search (Figure 2). Unfortunately, the Streaming API connection had a problem around that day, resulting in no data from the stream during that time. We compared tweets posted on 31 May and retrieved from PowerTrack and the Search APIs. The hourly volumes of tweets collected by the two APIs are displayed in Figure 3. The number of hourly tweets of PowerTrack data ranged from approximately 1200 to 3100; on the contrary, the number of tweets from the Search API was rather steady and did not exceed 1600, overlapping with the PowerTrack data from 40% to 90% (Figure 3). It appears the Search API is less likely to detect spikes and thus unsuitable to observe trends over time.

Content: The top 10 hashtags on both of the PowerTrack data and the Search data and the proportion of tweets that mentioned the hashtags are presented in Table 1. Same hashtags were found as the most frequent 10 between the two APIs, although their orders are slightly different. Kendall’s tau to measure the correlation of ordered hashtags was 0.84. These results suggest that the two APIs share main contents.

### 3.3. E-Cigarette

**Figure 4:**
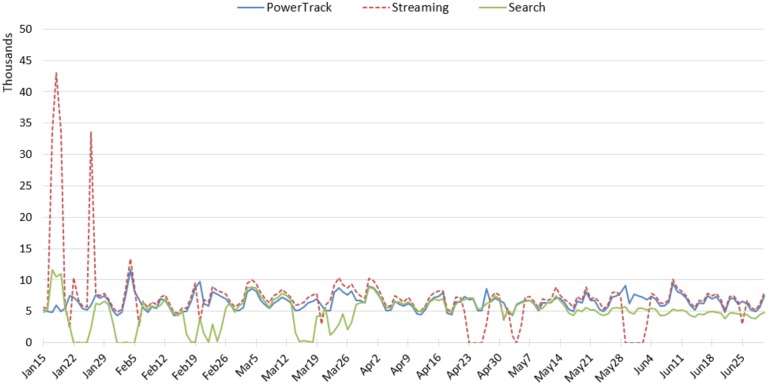
Daily amount of e-cigarette-related tweets across the APIs.

Amount: Similar to the tobacco topic, the Streaming API retrieved the most e-cigarette tweets on most days, followed by PowerTrack (Figure 4). The Streaming and the Search APIs had multiple interruptions. The Streaming retrieved excessive daily counts on 17–19 January and 26 January, which were not captured by PowerTrack. The Search partially captured the spike on 17–19 January and had an issue when the second spike occurred on the 26th.

**Figure 5:**
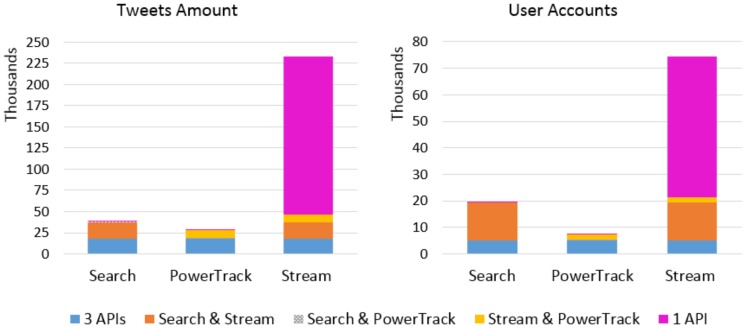
Number of e-cigarette tweets and associated accounts in January 2015. Blue indicates the number of tweets retrieved by all three APIs, yellow, textured gray, and orange indicate the amount retrieved by two APIs, and magenta the amount of tweets unique to each API.

We further inspected the e-cigarette-related tweets posted in January. Figure 5 presents the number of tweets and user accounts retrieved by the three APIs. The Streaming API captured significantly more tweets and accounts: Streaming API retrieved about 8 times more than PowerTrack, and those tweets were posted by about 10 times more accounts than PowerTrack. In addition, 80% of the tweets (n = 186,424) and 71% of the accounts (n = 53,033) collected by the Streaming were unique to that API. This result contradicted our hypothesis, as well as findings of existing literature. Although it is not clearly visible in the Figure 5, both of the Search and PowerTrack retrieved small numbers of unique tweets and accounts as well.

During the spikes in January, the Streaming and Search APIs retrieved 122,987 tweets the PowerTrack did not retrieve. Of those, the top frequent tweet (88%) was “rt @app******mko: [Japanese text + multiple URLs]”. This particular tweet was an advertisement of a role-playing game app and included a few URLs, one of which included the string “/ecig/”. The account @app******mko has been suspended possibly because it was recognized as a spammer. Further, this tweet was posted by a large number of different accounts, with many posted at the very same time--to the second--exhibiting bot-like behavior. The second most frequent tweet (9%) was “rt @vap******net: do you know that you can buy Hangsen e-liquids with free delivery? Check [URL]”. This message was also posted by many different accounts, of which many were suspended or deactivated. The third most frequent tweet (0.6%) was “rt @va***_b: Mignon Box Mod ! #ecig [URL]”. This suggests the Streaming (and the Search) API may have retrieved substantially more marketing-related and bot-generated tweets.

User Account: We further inspected differences in user accounts of tweets by labeling 1000 randomly sampled accounts. Table 2 shows the number of accounts labeled for e-cigarette relevance, primary language, and marketing. There were significant differences in e-cigarette relevance and English as the primary language across the accounts retrieved from each API. These differences suggest that the data retrieved by the two public APIs may require more careful data filtering/cleaning. To count marketing accounts, we restricted the data to user accounts that posted e-cigarette-relevant tweets in English. This removed the retweets advertising the game app. The Streaming API shows slightly more e-cigarette marketing accounts although the difference is not statistically significant.

More importantly, of the e-cigarette marketing accounts, we found that 148 (56.1%) from the Streaming API were suspended (15.2%) by Twitter or deleted (40.9%) by users themselves, 37 (31.3%) from the Search API were suspended (11.0%) or deleted (20.3%), and 103 (27.4%) from the PowerTrack were suspended (8.7%) or deleted (18.7%) before the time of writing this manuscript.

### 3.4. Anti-Smoking

**Figure 6:**
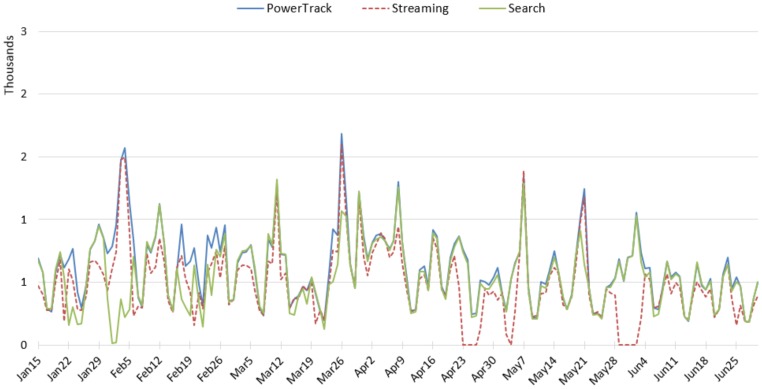
Daily amount of anti-smoking-related tweets across APIs.

Amount: PowerTrack retrieved the most anti-smoking tweets, closely followed by the Search API; the Streaming API retrieved the fewest (Figure 6). However, the differences between the three APIs are relatively small for the anti-smoking topic, compared to the other two topics. On multiple days, the Streaming API showed much lower counts, even when there was no interruption in the API connection. Because of its relatively small amount, this anti-smoking topic was less affected by the API connection problem than the other topics.

## 4. Discussion

We conducted an experiment to compare three widely-used access points of Twitter data—Streaming API, Search API, and Historic PowerTrack API—for studying three topics that represent different levels of tweeting activity—tobacco, e-cigarette, anti-smoking. For each source and topic, we examined the amount, content, and user accounts of tweets retrieved using the same keywords and study period. While we expected the Historic PowerTrack to serve as a gold standard, retrieving the most data across all three topics, we found that the Streaming API retrieved the most tweets for tobacco and e-cigarette topics. In particular, the vast majority of e-cigarette-related tweets collected in January via the Streaming was not captured by either PowerTrack or the Search API. The Historic PowerTrack retrieved more for the small-volume topic, anti-smoking. Beyond discrepancies in the amount of retrieved posts across APIs, we also found that the content and accounts of retrieved tweets varied substantially. In particular, Gnip’s Historic PowerTrack did not retrieve the large volume of marketing and advertising tweets, including e-cigarette marketing that peaked around the New Year, when many cigarette smokers aspire to quit.

Also notable was the relatively large number of irrelevant tweets retrieved by the Streaming API: post-processing of the data retrieved from the Streaming API revealed that more than a million tweets did not in fact match any of our keywords in the tweet text or embedded URLs. Others have observed that because the Streaming API does not support a sophisticated search filter, it is likely to retrieve more irrelevant data that should be excluded after the data collection is completed [26]. Our results confirm this and suggest that data collected via the Streaming API require more careful data filtering and cleaning processes prior to analysis.

While the Streaming API had relatively poor retrieval precision [8], the data retrieved from the Search API suffered from poor retrieval recall when no API connection problem occurred. The Search API can accommodate more complicated search filters than the Streaming API, and can execute separate searches for each keyword, but this process can increase the number of files generated and risk of encountering rate limits, resulting in a truncated dataset.

Further, our experiments showed that sporadic connectivity problems and rate limits can also severely truncate the data. For example, our analysis of tobacco-related tweets posted on World No Tobacco Day illustrated that the Search API appeared to hit the rate limit on this important day for tobacco surveillance. Coincidentally, the connection with the Streaming API was unstable that same day. Thus, each of these publicly available sources of Twitter data may be unsuitable for surveillance purposes. Indeed, Twitter’s own documentation of the Search API suggests that the Streaming API provides more complete data [30]. However, the Streaming API has its own limitations. In addition to the risk of data loss due to connectivity lapses, it is particularly challenging to construct comprehensive search rules to retrieve conversations around a topic of interest in real time and sometimes nearly impossible to set up queries in anticipation of a viral incident or organic cultural moment [31]. In addition, there is currently no way to distinguish which keyword retrieved which tweets with the Streaming API that would help data collection and cleaning. Joint collection with both APIs could partially compensate for drawbacks of each, but requires doubling resources for data collection and management.

The Historic PowerTrack provides access to an archive of the full stream of publicly available Twitter data, known as the Firehose. While the terms of service specify that deleted tweets and accounts cannot be accessed via the Historic PowerTrack, little is known about the volume, content or user accounts of deleted tweets—whether user-deleted or from accounts that Twitter has suspended. Our experiment showed that the majority of unique tweets retrieved by the Streaming API was related to suspended or deactivated accounts; they were either posted by those accounts or retweeted mentions of those accounts. For example, the original tweet that promoted Hangsen e-liquids can be still found via PowerTrack, but the more than 11,000 retweets generated by many (bot-like) accounts are not anymore available in PowerTrack. Further, we observed more than half the sample of user accounts related to e-cigarette marketing from the Streaming API have been deleted or suspended since the time of data retrieval. We do not know how influential those accounts and tweets were in the promotion of that particular product, but we do know that most tweets are viewed as they stream or relatively close to the time they are posted [32,33,34] and that online information exposure influences offline behavior [35,36,37]. Thus, it is possible that tweets that were deleted after they were originally posted (and captured by the Streaming API) influenced the behavior of a large audience prior to deletion. While Twitter acknowledges the value of preserving and providing access to public records [38], it is currently impossible to retrospectively analyze the impact of tweets that were deleted or posted from subsequently suspended accounts. The Premium Search API that Twitter recently launched returns counts for a specified query including deleted ones, however it still does not allow to explore the content of those deleted.

This pilot study has a few limitations. We experienced multiple interruptions with the Streaming and Search APIs connections that resulted in no or minimal data collected during the disconnections. The interruptions may have occurred due to a combination of issues: maintenance of the server that ran scripts requesting data pull/push, the streaming server overload, network congestion, and unknown glitches. We suspect that server maintenance was probably the main reason we experienced the interruptions, although we did not have a system in place for constant monitoring of the API connections. The lack of a monitoring system limited our analysis to a subset of the APIs in some cases. Use of a cloud server may alleviate the issue with a modest cost. Our experience is likely not uncommon, and strongly suggests that a system of constant monitoring and documentation of any issues and repairs is necessary with the public APIs. Otherwise, problems can go unnoticed. Our Twitter data collection occurred in 2015, and current techniques and constraints of Twitter APIs may differ from 2015. However, our findings still speak to the importance of understanding and disclosure of data sources.

We compared tobacco-related tweets collected via the popular three APIs. Our topics were broad enough to observe the limitations of Firehose data, but not general enough to capture high activity that goes beyond the Streaming API’s 1% limit. A study that explores a more general topic, beyond health-related data collection, which is supported by an infrastructure that minimizes potential problems with API connections is needed to better understand the limitation and generalize the findings.

## 5. Conclusions

Our study found that Historic PowerTrack data may not contain certain types of tweets and accounts. Specifically, it may underestimate the amount of marketing and bot-generated tweets and accounts. This is a unique contribution of our study. Researchers should be cautious about using PowerTrack data to study marketing-related and bot-generated contents and accounts. However, in the current media ecosystem, where such accounts potentially disrupt social discourse and affect real-world events, it is crucial to understand the amount, reach, and impact of such messages.

Our research underscores the importance of clearly understanding, evaluating, and describing the advantages and limitations of data used for any social media studies. Our work highlights the value of reporting data sources, data quality, and analytic data preparation for transparency and replicability. Basic principles to assure quality of traditional data apply to social media data too. A clear disclosure and understanding of all processes involved in data collection, cleaning, and management should be strongly encouraged in the social media research community.

## Figures and Tables

**Table 1:** Top hashtags of tobacco-related tweets on World No Tobacco Day.

Hashtags	PowerTrack (%)	Search API (%)
#NoTobacco	6.3	6.1
#WorldNoTobaccoDay	2.5	2.3
#SayNoToTobacco	2.2	2.0
#tobacco	1.7	1.8
#vape	1.1	1.2
#ecig	0.9	1.1
#TobaccoKillsEvenNinjas	0.9	0.6
#WNTD2015	0.7	0.7
#vaping	0.5	0.4
#NoTobaccoDay	0.5	0.5

**Table 2:** Random samples of Twitter user accounts from each of the APIs.

	PowerTrack (*n* = 1000)	Search (*n* = 1000)	Streaming (*n* = 1000)
E-cigarette Relevant	983	309	586
Post in English	912	288	570
E-cigarette Relevant & in English	900	285	563
- Marketing, *N* (%)	380 (42.2%)	118 (41.4%)	264 (47.0%)

## Appendix A

**Table A1:** Monthly volume of overlapping tweets between the APIs, combining all three topics.

		Jan	Feb	Mar	Apr	May	Jun	Total
3 APIs ^1^	All	220,862	315,063	458,789	542,922	446,477	448,688	2,432,801
2 APIs ^2^	Search & Stream	38,663	33,881	59,103	64,452	66,745	44,605	307,449
	Search & PowerTrack	14,676	17,967	18,166	130,372	145,965	92,041	419,187
	Stream & PowerTrack	174,629	390,600	282,540	70,953	100,267	148,189	1,167,178
1 API ^3^	Search	1871	3294	5362	19,290	24,333	15,078	69,228
	PowerTrack	30,735	49,995	28,527	37,106	67,935	37,965	252,263
	Stream	145,702	91,744	86,400	47,546	194,437	189,115	754,944
Month Total		627,138	902,544	938,887	912,641	1,046,159	975,681	5,403,050

^1^: Overlapping tweets returned by all three APIs; ^2^: Overlapping tweets returned by pairs of two APIs; ^3^: Unique tweets returned by each of the three APIs.
